# Food Insecurity and Its Sociodemographic Correlates among Afghan Immigrants in Iran

**DOI:** 10.3329/jhpn.v31i3.16828

**Published:** 2013-09

**Authors:** Nasrin Omidvar, Mahmoud Ghazi-Tabatabie, Rasoul Sadeghi, Fatemeh Mohammadi, Mohammad Jalal Abbasi-Shavazi

**Affiliations:** ^1^Department of Community Nutrition, Faculty of Nutrition Sciences and Food Technology, Shahid Beheshti University of Medical Sciences, Tehran, Iran; ^2^Department of Demography, Faculty of Social Science, University of Tehran, Iran; ^3^Department of Demography, University of Tehran, Iran and Australian Demographic and Social Research Institute, Australian National University, Canberra, Australia

**Keywords:** Afghan, Immigrants, Food insecurity, Sociodemographic determinants, Iran

## Abstract

The study determined the prevalence of food insecurity and its sociodemographic determinants among Afghan immigrants in two major cities of Iran. This cross-sectional study was conducted on a sample of 310 adult females from immigrant Afghan households in Tehran (n=155) and Mashhad (n=155), who were recruited through multistage sampling. Data were collected through face-to-face interviews, using a questionnaire. Food security was measured by a locally-adapted Household Food Insecurity Access Scale. More than 60% suffered from moderate-to-severe food insecurity, 37% were mildly food-insecure while about 23% were food-secure. Food insecurity was significantly more prevalent in female-headed households, households whose head and spouse had lower level of education, belonged to the Sunni sect, and those with illegal residential status, unemployment/low job status, not owning their house, low socioeconomic status (SES), and living in Mashhad. Prevalence of food insecurity was relatively high among Afghan immigrants in Iran. This calls for the need to develop community food security strategies for ensuring their short- and long-term health.

## INTRODUCTION

In the history of the modern world, the situation in Afghanistan has resulted in one of the largest displacements of mankind in search of safety and a better life (1). Migratory movements of the Afghans to Iran have a long history. Transitory migration of the Afghans to Iran motivated by economic differences has occurred since the nineteenth century. Also, Shiite Afghans have been making pilgrimages to Iran for several hundred years. However, the modern history of Afghan immigration to Iran started in 1979. Since then, Afghan immigration to Iran has been primarily motivated by the direct and indirect effects of war, insecurity, threat to female honour (*namoos*), unemployment, and inflation. In this process, Iran, as one of the most concentrated destination of immigrants and refugees, has hosted around 3 million Afghan migrants and refugees in the past three decades. In spite of the repatriation programme, still around 2 to 2.5 million Afghan immigrants, both legal and illegal, live in Iran (2-4).

Health and nutrition status of immigrants is of concern from public-health point of view. Food insecurity that is characterized by “limited access to or availability of nutritionally-adequate, culturally-relevant, and safe food and/or limited or uncertain ability to acquire food in socially acceptable ways” (5), has long been a problem for the most vulnerable and under-represented segments of the population, including foreign immigrants and refugees. However, research in this field is rare and, thus, the need for assessing the extent of food insecurity among such vulnerable and hard-to-reach segments of the population is of great importance (6).

Food insecurity represents a major public-health concern and is a useful index of health and well-being because it is associated with poverty, ill-health, poor dietary intake (e.g. low intake of fruits and vegetables), limited social capital, and depressive disorders (7). The dynamics of food insecurity reflect the composition and financial circumstances of families (8). In case of immigrants, different factors are identified as being responsible for food insecurity, including economic constraints posed by poverty, low-wage employment, job insecurity, education, and marginal social position as well as the obligation to send money to family remaining in their country of origin in some cases. Also, many immigrants face additional challenges due to lack of valid immigration documents and fear of being deported. Therefore, immigrants are distinguished from many non-immigrants because they face additional pressure that may strain already-limited household food resources. Based on results of previous studies, immigrant families are more likely than native families to face food insecurity. Research also suggests that food insecurity is higher among less-acculturated immigrants (9-10).

Despite the large volume and long trend of Afghan migratory movements to Iran, little information is available on nutritional status of Afghan immigrants in Iran (11). Thus, the present study was undertaken to examine the extent of food insecurity and its correlates among Afghan immigrants in two metropolitan cities Tehran and Mashhad in 2010.

## MATERIALS AND METHODS

### Study design

This cross-sectional study was conducted in the framework of a larger study on the adaptation of Afghan immigrants in Iran (3). The study was performed in Tehran and Mashhad two metropolitan cities of Iran during February and March 2010. Based on 2006 Census, the majority of Afghan immigrants (72%) resided in urban areas of the country while only less than 3% were in refugee camps. The major provinces that host these immigrants include Tehran (32.7%), Khorasan Razavi (Mashhad) (13.3%), Isfahan (11.7%), and Sistan-Baluchistan (9.3%) (3). Selection of Tehran and Mashhad as the study sites was due to the sizeable population of Afghans in these two cities.

### Subjects

A sample of 310 adult females (aged 20 years and above) from Afghan households residing in Tehran (n=155) and Mashhad (n=155) was recruited. Multistage sampling was done through a two-stage procedure. In the first stage, stratified cluster-sampling procedure was used for selecting the location and blocks with high concentration of the Afghans in each city. In the second stage, chain-referral (snowball) sampling technique was used. Drawing a random sample of immigrants is not possible; thus, snowball sampling technique is the most widely-applied method of survey among immigrants.

The respondents were chosen from married women (housewives) who were directly involved in food preparation in the households.

### Data collection

Data were collected through face-to-face interviews by six trained Afghan female interviewers who were college students or graduates. The interviewers were trained prior to data collection in a one-day workshop conducted by the research team at the Faculty of Social Science in the University of Tehran. Through this workshop, the interviewers were trained on the purpose of the study, sampling procedures, considerations in administration of the questionnaire, and other field-related factors.

Data on sociodemographic characteristics, including age, sex, level of education, and occupational status of household head and spouse as well as family-size, expenditure, residency, and living conditions, were gathered through a structured questionnaire.

Household socioeconomic status (SES) was defined by composite indicators, including household assets (housing material, car, motorcycle, computer, colour television, freezer, vacuum cleaner, washing machine, and furniture) and average monthly expenditure per capita. We used principal component analysis (PCA) to construct the SES score and, based on the range of the score, the households were categorized into three SES categories: high, middle, and low.

In addition, socioeconomic position of the neighbourhood was also categorized into three categories: high, middle, and low, based on socioeconomic level of the area where the subjects resided. Neighbourhood index of socioeconomic development was determined using information from the 2006 Census in Iran on average education and occupational attainment by residents of that neighbourhood and their access to facilities.

### Measurement of food insecurity

Food security was measured at the household level. A locally-adapted HFIAS (Household Food Insecurity Access Scale) developed by USAID's Food and Nutrition Technical Assistance (FANTA) project was used in measuring food security in each household. The scale consisted of 9 items with 4 frequency options that classified households into secure, mild, moderate, and severe food-insecure. Steps taken in adaptation and validation of the scale is presented elsewhere (12).

### Statistical analysis

Data were analyzed by SPSS software (version 16.0). Descriptive statistics were used in examining the association between food security status and each of the independent variables, using analysis of variance and χ^2^-test.

The likelihood of being food-insecure was determined by regression analyses, using binary logistic regression. Also, multinomial logistic regression was used in estimating the association between selected household characteristics and food security status. In this case, households were classified as ‘food-secure’, ‘mild-to-moderately food-insecure’, and ‘severely food-insecure’.

## RESULTS

### Characteristics of the sample

The respondents included 310 Afghan women with mean age of 35.3±11 years, who were either head of the household (n=25), or spouse (n=278) and, in a few cases (n=7), older unmarried girls in the household. The characteristics of respondents and their households are displayed in Table 1.

### Household food insecurity status

Distribution of responses to the nine questions in HFIAS is presented in Table 2. More than 40% of women reported worrying that food would run out while 16% had experienced actual lack of any food within the household, and less than 10% had gone to sleep hungry more than once. As shown in the figure, more than 60% Afghan households included in the study suffered from moderate-to-severe food insecurity while only 23% were food-secure.

### Food insecurity and sociodemographic correlates

Bivariate analysis showed no significant association between the age of respondent as well as age of the household head and food security status while food security status was significantly correlated with the duration of residency in Iran, average household monthly expenditure per capita, and SES (Table 3).

**Table 1.** Sociodemographic characteristics of Afghan respondents, 2010

Sociodemographic characteristics	Frequency	Percentage	Mean±SD
Age (years)			
15-<30	123	39.7	35.3±11.2
30-<45	112	36.1	
≥45	75	24.2	
Marital status			
Married	280	90.3	
Widow	23	7.4	
Unmarried	7	2.3	
Educational levels			
No education	120	38.7	
Primary	117	37.7	
Secondary	32	10.3	
Diploma and higher	41	13.2	
Occupational status			
Unemployed	287	92.6	
Employed	23	7.4	
Religion			
Shiite	225	72.6	
Sunni	85	27.4	
Birthplace			
Afghanistan	244	78.7	
Iran	66	21.3	
Duration of residency in Iran (years)			
<10	42	13.5	22.9±10.2
10-<20	72	23.2	
20-<30	122	39.4	
≥30	74	23.9	
Legal status			
Documented	245	79.0	
Undocumented	65	21.0	
City			
Tehran	155	50.0	
Mashhad	155	50.0	

There was no relationship between household food insecurity and marital status, birth place, and city of residency (Table 4). However, prevalence of food insecurity significantly increased with increasing household-size, in female-headed households, those households whose head and spouse had lower educational level, those belonging to the Sunni sect, those with illegal residential status, unemployed/low job status, those who did not own their house, and low SES, and households in poor neighbourhoods. Food insecurity decreased with increase in the duration of residency in Iran and the level of household SES.

**Table 2.** Responses to nine questions in HFIAS applied among Afghan households in Iran, 2010

HFIAS questions	Options			
	No	Rarely	Sometimes	Often
Q1: Worry about food	185 (59.7)	56 (18.1)	29 (9.4)	40 (12.9)
Q2: Unable to eat preferred foods	102 (32.9)	70 (22.6)	77 (24.8)	61 (19.7)
Q3: Eat just a few kinds of foods	109 (35.2)	51 (16.5)	83 (26.8)	67 (21.6)
Q4: Eat foods that really do not want to eat	104 (33.5)	71 (22.9)	80 (25.8)	55 (17.7)
Q5: Eat a smaller meal	217 (70.0)	53 (17.1)	25 (8.1)	15 (4.8)
Q6: Eat fewer meals in a day	254 (81.9)	28 (9.0)	21 (6.8)	7 (2.3)
Q7: No food of any kind in the household	261 (84.2)	34 (11.0)	14 (4.5)	1 (0.3)
Q8: Go to sleep hungry	280 (90.3)	21 (6.8)	7 (2.3)	2 (0.6)
Q9: Go a whole day and night without eating	304 (98.1)	4 (1.3)	2 (0.6)	0 (0.0)
Figures in parentheses indicate percentages				

**Figure.**
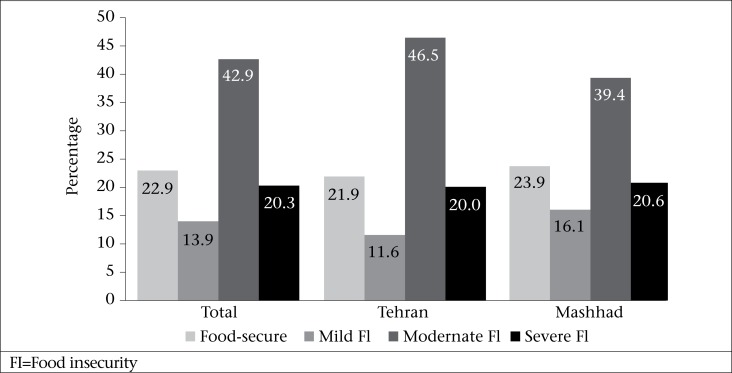
Food insecurity among Afghan households in Tehran and Mashhad, 2010

Binary and multinomial logistic regression analyses were used in investigating the association of demographic and socioeconomic factors with food insecurity. Table 5 presents the estimated odds ratios (OR) and 95% CI of binary logistic regression analysis. The dependent variable is a dichotomous measure of food security versus food insecurity. The model was initially fitted with fifteen factors, of which nine were found to be significantly associated with food insecurity. These included educational level of respondent, age and gender of household head, type of job, household monthly expenditure, housing tenure, SES, economic situation of the neighbourhood, and city context. No significant differences were observed in the prevalence of food insecurity by occupational status, religion, birthplace, legal status, household-size, and the duration of residency in Iran.

As shown in Table 5, educational level was a protective factor against food insecurity. The probability of household food security was higher when household heads had a high school diploma and higher level of education compared to those with lower educational level. Also, labourers and farmers had more prevalence of food insecurity compared to persons with salaried occupations. With increase in age of the household head, the likelihood of being food-insecure increased. Female-headed households were more likely to be food-insecure than households headed by males.

The probability of food insecurity in the households with rented house was higher than those who owned their house. The prevalence of food insecurity was significantly higher in households in poor neighbourhoods than in those with high SES. In addition, the result showed that food insecurity among Afghan households in Mashhad was high compared to those in Tehran.

**Table 3.** Association between some sociodemographic variables and food security status among Afghans in Iran, 2010

Variable	Food security status (Mean±SD)			
	Food security (n=71)	Mild insecurity (n=43)	Moderate insecurity (n=133)	Severe insecurity (n=63)
Age of respondent (years)	33.6±11.2	34.6±11.6	36.2±10.9	35.8±11.8
Age of head of household (years)	40.1±12.8	42.0±14.3	42.6±12.7	43.2±14.2
Household-size (numbers)*	5.1±2.0	6.3±2.7	6.0±2.5	6.6±2.6
Duration of residency in Iran (years)***	26.2±8.1	22.3±9.2	22.5±10.7	20.4±11.3
Average household monthly expenditure per capita (US$)***	125±98.6	67.3±40.8	64±31.8	55.8±33.2
Score of socioeconomic status**	59.6±10.4	49.7±8.0	46.7±8.3	44.2±8.9

*p<0.05,

**p<0.01,

***p<0.001

Multinomial logistic regression was used in estimating the association between selected individual/household characteristics and household food security status. In this analysis, we classified household food security level as ‘food-secure’, ‘mild-to-moderately food-insecure’, and ‘severely food-insecure’. Results of multinomial logistic regression analyses are displayed in Table 6. The first set of coefficients predicts severe food insecurity versus food security, the second set of coefficients compares mild-to-moderate food insecurity versus food security, and the third set of coefficients compares severe versus mild-to-moderate food insecurity.

Compared to food-secure households, low level of education, being Sunni, labourer, and farmer, having older age, and female gender of the household head, having less than US$ 50 monthly household expenditure per capita, having renting house, belonging to low social class, living in poor neighbourhood, and living in Mashhad increased the risk of severe food insecurity. In turn, living in Iran for 20-30 years decreased this risk.

When comparing mild-to-moderately food-insecure households with food-secure ones, the major predictors of food insecurity included low educational level, older age, and female gender of the household head as well as less than US$ 50 monthly expenditure per capita, having renting house, low-to-middle SES, living in neighbourhood with poor-to-middle SES, and living in Mashhad.

**Table 4.** Frequency of different levels of food insecurity based on socioeconomic variables in Afghan households in Tehran and Mashhad, 2010

Variable	Food security	Mild insecurity	Moderate insecurity	Severe insecurity
Head of household*				
Male (n=285)	70 (24.6)	41 (14.4)	118 (41.4)	56 (19.6)
Female (n=25)	1 (4.0)	2 (8.0)	15 (60.0)	7 (28.0)
Marital status				
Married (n=280)	69 (24.6)	41 (14.6)	116 (41.4)	54 (19.3)
Widowed (n=23)	1 (4.3)	1 (4.3)	14 (60.9)	7 (30.4)
Unmarried (n=7)	1 (14.3)	1 (14.3)	3 (42.9)	2 (28.6)
Educational levels**				
No education (n=120)	14 (11.7)	15 (12.5)	58 (48.3)	33 (27.5)
Primary (n=117)	28 (23.9)	19 (16.2)	49 (41.9)	21 (17.5)
Secondary (n=32)	12 (37.5)	2 (6.3)	14 (10.5)	4 (12.5)
Diploma and higher (n=41)	17 (41.5)	7 (17.1)	12 (29.3)	5 (12.2)
Occupational status*				
Unemployed (n=287)	66 (23.0)	35 (12.2)	125 (43.6)	61 (21.3)
Employed (n=23)	5 (22.7)	8 (34.8)	8 (34.8)	2 (8.7)
Religion**				
Shiite (n=225)	58 (25.8)	33 (14.7)	99 (44.0)	35 (15.6)
Sunni (n=85)	13 (15.3)	10 (11.8)	34 (40.0)	28 (32.9)
Birthplace*				
Afghanistan (n=244)	52 (21.3)	34 (13.9)	107 (43.9)	51 (20.9)
Iran (n=66)	19 (28.8)	9 (13.6)	26 (39.4)	12 (18.2)
Duration of residency in Iran (years)**				
<10	5 (11.9)	7 (16.7)	19 (45.2)	11 (26.2)
10-<20	8 (11.1)	10 (13.9)	30 (41.7)	24 (33.3)
20-<30	38 (31.1)	17 (13.9)	54 (44.3)	13 (10.7)
≥30	20 (27.0)	9 (12.2)	30 (40.5)	15 (20.3)
Legal status*				
Documented (n=245)	63 (25.7)	35 (14.3)	103 (42.0)	44 (18.0)
Undocumented (n=65)	8 (12.3)	8 (12.3)	30 (46.2)	19 (29.2)
Educational levels of husband*				
No education (n=58)	7 (12.1)	9 (15.5)	29 (50.0)	13 (22.4)
Primary (n=101)	19 (18.8)	17 (16.8)	45 (44.6)	20 (19.8)
Secondary (n=57)	20 (35.1)	6 (10.5)	22 (38.6)	9 (15.8)
Diploma and higher (n=64)	23 (35.5)	9 (14.1)	20 (31.3)	12 (18.8)
Occupation of husband**				
Unemployed (n=14)	2 (14.3)	3 (21.4)	6 (42.9)	3 (21.4)
Labourer, Farmer (n=88)	8 (9.1)	16 (18.2)	42 (47.7)	22 (25.0)
Freelancer, Shopkeeper (n=157)	47 (29.9)	19 (12.1)	63 (40.1)	28 (17.8)
Employee, Teacher, Clergy (n=12)	5 (41.7)	3 (25.0)	4 (33.3)	0 (0)
Doctor, Employer (n=5)	5 (100.0)	0 (0)	0 (0)	0 (0)
Housing tenure**				
Ownership (n=66)	26 (39.4)	10 (15.2)	23 (34.8)	7 (10.6)
Renting (n=244)	45 (18.4)	33 (13.5)	110 (45.1)	56 (23.0)
Socioeconomic status of household***				
Low (n=89)	5 (5.6)	10 (11.2)	46 (51.7)	28 (31.5)
Middle (n=182)	35 (19.2)	30 (16.5)	83 (45.6)	34 (18.7)
High (n=39)	31 (79.5)	3 (7.7)	4 (10.3)	1 (2.6)
Socioeconomic position of location***				
Low (n=143)	15 (10.5)	21 (14.7)	71 (49.7)	36 (25.2)
Middle (n=109)	27 (24.8)	15 (13.8)	52 (47.7)	15 (13.8)
High (n=58)	29 (50.0)	7 (12.1)	10 (17.2)	12 (20.7)
City				
Tehran (n=155)	34 (21.9)	18 (11.6)	72 (46.5)	31 (20.0)
Mashhad (n=155)	37 (23.9)	25 (16.1)	61 (39.4)	32 (20.6)

*p<0.05

**p<0.01

***p<0.001

Figures in parentheses indicate percentages

**Table 5.** Sociodemographic factors relating to the likelihood of being food-insecure among Afghan households in Tehran and Mashhad, 2010 (The results of binary logistic regression)

Sociodemographic factor	Likelihood of being food-insecure		
	B	Exp (B) (Odds ratio)	95% CI for Exp (B)
Intercept	-8.672***	-	-
Educational levels (Ref. Diploma to higher)			
No education	1.846**	6.332	(1.661-24.135)
Primary	1.882***	6.566	(1.834-23.508)
Secondary	0.865	2.376	(0.539-10.472)
Occupational status (Ref. Employed)			
Unemployed (Housewife)	-0.400	0.670	(0.124-3.632)
Religion (Ref. Shiite)			
Sunni	0.411	1.509	(0.460-4.952)
Birthplace (Ref. Iran)			
Afghanistan	-1.264	0.282	(0.070-1.133)
Legal status (Ref. Legal/documented)			
Illegal/undocumented	0.351	1.420	(0.315-6.398)
Job title of husband (Ref. Employee)			
Unemployed	0.472	1.604	(0.109-23.530)
Labourer, Farmer	1.693*	5.438	(1.032-31.765)
Freelancer, Shopkeeper	0.938	2.555	(0.471-13.877)
Age of household head (Ref. 15-<30 years)			
30-<45	0.973	2.645	(0.706-9.970)
45-<60	1.939**	6.952	(1.411-34.240)
≥60	2.406*	11.084	(1.048-121.63)
Gender of household head (Ref. Male)			
Female	2.148†	8.568	(0.899-245.77)
Household-size (Ref. 1-3)			
4-6	-0.774	0.461	(0.100-2.128)
7 and more	0.770	2.159	(0.566-8.233)
Duration of residency with family in Iran (Ref. ≥30 years)			
<10	0.389	1.475	(0.226-9.626)
10-<20	0.339	1.404	(0.299-6.586)
20-<30	-0.831	0.436	(0.148-1.286)
Household monthly expenditure per capita (Ref. More than US$ 100)			
Less than US$ 50	2.565***	13.007	(2.007-84.280)
US$ 50−75	0.300	1.350	(0.328-5.550)
US$ 76-100	0.258	1.295	(0.325-5.155)
Housing tenure (Ref. Ownership)			
Renting	2.102**	8.179	(2.198-30.442)
Socioeconomic status of household (Ref. High)			
Low	2.688***	14.707	(2.233-96.861)
Middle	2.201***	9.034	(2.367-34.476)
Socioeconomic position of location (Ref. High)			
Low	3.405***	30.116	(6.290-144.184)
Middle	2.003**	7.409	(2.367-34.476)
City area (Ref. Tehran)			
Mashhad	2.678***	14.557	(3.818-55.502)
-2 Log likelihood	178.03	Nagelkerke R-square	60.1%
Model chi-square (df)	155.6(28)***	Percent correctly predicted	85.5

†p<0.10

*p<0.05

**p<0.01

***p<0.001

B=Coefficient(s) on the independent variable(s)

df=Degree of freedom

Ref=Reference group

**Table 6.** Odds ratios (from multivariate analysis) of independent predictors of food insecurity in Afghan households in Tehran and Mashhad, 2010

Sociodemographic factor	Likelihood of being food-insecure					
	Severe FI vs Secure		Mild-to-moderate FI vs Secure		Severe FI vs Mild-to-moderate FI	
	B	Exp (B) (Odds ratio)	B	Exp (B)(Odds ratio)	B	Exp (B) (Odds ratio)
Intercept	-27.871***	-	-8.538***	-	-19.333***	-
Educational levels (Ref. Diploma to Up)						
No education	2.449***	11.577	1.722**	5.594	0.727	2.070
Primary	2.225*	9.256	1.851**	6.367	0.374	1.454
Secondary	0.622	1.862	0.835	2.305	-0.213	0.808
Occupational status (Ref. Employed)						
Unemployed (Housewife)	0.632	1.883	-0.530	0.588	1.152†	3.166
Religion (Ref. Shiite)						
Sunni	1.190*	3.288	0.235	1.265	0.955**	2.599
Birthplace (Ref. Iran)						
Afghanistan	-1.770*	0.172	-1.199	0.301	-0.570	0.565
Legal status (Ref. Legal/documented)						
Illegal/undocumented	0.506	1.658	0.385	1.469	0.121	1.129
Job title of husband (Ref. Employed)						
Unemployed	1.117*	3.056	0.416	1.516	0.445	1.560
Labourer, Farmer	1.792***	6.001	1.472†	4.359	1.693**	5.436
Freelancer, Shopkeeper	1.041	2.832	0.712	2.038	0.858	2.352
Age of household head (Ref. 15-<30 years)						
30-<45	0.531	1.701	1.023†	2.781	-0.492	0.611
45-<60	1.635†	5.130	1.996**	7.360	-0.361	0.697
≥60	2.672*	14.470	2.369*	10.687	0.303	1.354
Gender of household head (Ref. Male)						
Female	2.571*	13.081	1.979†	7.237	0.592	1.807
Household-size (Ref. 1-3)						
4-6	-1.021	0.360	-0.753	0.471	-0.267	0.765
7 and more	0.777	2.174	0.756	2.129	0.021	1.021
Duration of residency with family in Iran (Ref. ≥30 years)						
<10	0.404	1.498	0.463	1.589	-0.867	0.420
10-<20	0.182	1.201	0.301	1.351	-0.118	0.888
20-<30	-1.966**	0.140	-0.680	0.507	-1.286*	0.276
Household monthly expenditure per capita (Ref. More than US$ 100)						
Less than US$ 50	3.652***	38.616	2.399**	11.008	1.265*	3.543
US$ 50−75	0.092	1.097	0.355	1.426	-0.263	0.769
US$ 76-100	0.524	1.690	0.269	1.308	0.256	1.291
Housing tenure (Ref. Ownership)						
Renting	2.764***	15.867	2.016**	7.509	0.748	2.113
Socioeconomic status of household (Ref. High)						
Low	3.528**	34.055	2.526***	12.505	1.002	2.723
Middle	2.795*	16.356	2.099**	8.154	0.696	2.006
Socioeconomic position of location (Ref. High)						
Low	2.841**	17.140	3.538***	34.382	-0.696	0.499
Middle	0.948	2.580	2.220***	9.211	-1.273*	0.280
City area (Ref. Tehran)						
Mashhad	3.119***	22.626	2.563***	12.978	0.556	1.743
-2 Log likelihood	403.23					
Pseudo R-square	56.2 %					
Model chi-square (df)	204.7(56)***					

†p<0.10

*p<0.05

**p<0.01

*** p<0.001

B=Coefficient(s) on the independent variable(s)

df=Degree of freedom

FI=Food insecurity

Ref=Reference group

Comparison of severely food-insecure households with mild or moderately food-insecure group showed that being Sunni, unemployed, labourer and farmer, and having less than US$ 50 monthly expenditure per capita increased the risk of severe food insecurity while living in Iran for 20-30 years and living in neighbourhood with middle SES decreased the risk.

## DISCUSSION

To the best of our knowledge, this is one of the first studies on food security status of Afghan refugees in Iran. The result indicated high prevalence of food insecurity among the studied households. The extent of food insecurity observed in this study is higher than those reported on immigrant Afghans in Pakdasht (11), which is a low-income city, located south of Tehran and considerably higher than estimates on Iranian households (12) as well as Latino immigrant families in the USA (13). However, food insecurity levels observed among Afghan immigrants in Iran are comparable with those of migrant and seasonal farm worker (MSFW) households on the USA-Mexico border (14). The Afghan immigrants face a high risk of food insecurity due to poverty, low human capital, limited access to resources and good job opportunities, and structural barriers to integration into Iranian society.

Analysis of influencing factors showed that immigrant households can be affected by food insecurity in different ways depending on their sociodemographic characteristics and economic situations. The major predictors identified for food insecurity in Afghan households in the present study included older age and female gender of household head, low educational level, being Sunni, low job status, low SES, having less than US$ 50 monthly expenditure per capita, having renting house, living in poor neighbourhood, and living in Mashhad. A recent study on determinants of food insecurity among Iranian households in the city of Tehran identified residency in medium- and low-SES districts, low education and job level of household head, lower income, expenditure, and facilities in the house as the major determinants of food insecurity among the studied population (12). In Pakdasht study, the low level of education of the household head and spouse (mother), sizeable number of young children in the family, and female gender of the head were also shown to be major predictors of household food insecurity (11). We have identified old age, belonging to the Sunni sect, living in a rented house and in Mashhad to be additional risk factors for food insecurity among Afghan refugees.

One of the important findings of the present study is that food insecurity was inversely associated with the length of stay in Iran. Previous studies had shown higher prevalence of household food insecurity among the recent immigrant households in Canada (6,15-16) and the USA (8-9). Longer length of residency has been shown to be related to increased acculturation, adaptation, and livelihood strategies.

Current evidence suggests the association between chronic household food insecurity and adverse health (17-19), including increased morbidity and mortality (20-22) and obesity (23-25) or underweight both in adults (26) and children (27). Although we could not measure health or nutritional status of the subjects, such findings re-emphasize the importance of social policy for immigrants in the country.

### Limitations

This study had several limitations; the small sample-size and lack of random sample selection could prevent generalization to the total population of Afghan immigrants in Iran. Though not ideal, convenience sampling was necessary because no sampling frame is available for this sensitive and hard-to-reach population. Also, the high level of food insecurity observed among the Afghans in this study could be partly due to the selectivity of the Afghans in Iran; all are labourers, with low level of SES.

Income data were not collected in the study because of the difficulty for immigrants to estimate incomes that vary monthly. Assessment of dietary intake or other indicators of nutritional status were not possible in the frame of the present study.

### Conclusions

This research provides new insights into the prevalence and factors associated with food insecurity among immigrant and refugee households in Iran.

Despite their longstanding presence of the Afghans in Iran, they remain excluded from key aspects of social, political and economic life in the country. The Afghans in Iran are employed mostly in jobs requiring little or no skills because of structural barriers and low human capital, and they are mostly concentrated in low socioeconomic settings. The prevalence of food insecurity among Afghan immigrants is unacceptably high. Afghan refugees and immigrants are a potentially vulnerable group, and our results suggest a need for more thorough monitoring of their health and well-being.

Policy-makers need to reconsider access to food programmes in light of the level of food insecurity and its short- and long-term consequences. Future studies are needed to evaluate and compare the results with those of the Afghan and Iranian counterparts. Also, further studies are needed to monitor levels of food insecurity in immigrant households and the strategies that they adopt for coping.

## ACKNOWLEDGEMENTS

This research was partially supported by Faculty of Social Sciences, University of Tehran under Grant No. 3105005-1-04. We thank the Afghan students in the University of Tehran and Mashhad, who conducted the interviews. Valuable comments from Tahmeed Ahmed are gratefully acknowledged.
